# Supplementary material for: Spatial variation in housing construction material in low- and middle-income countries: A Bayesian spatial prediction model of a key infectious diseases risk factor and social determinant of health
Source: PLOS Glob Public Health. 2024 Dec 18;4(12):e0003338. doi: 10.1371/journal.pgph.0003338 (PMC11654929; doi:10.1371/journal.pgph.0003338)
Supplement: S1 Text — (PDF) [file pgph.0003338.s001.pdf]

Supplementary Methods:

Spatial variation in housing construction material in low- and middle-income countries: a Bayesian spatial prediction model of a key infectious diseases risk factor and social determinant of health

1. *Exploratory variography*

Semi-variograms on Pearson residuals from non-spatial logistic regression models were constructed using the gstat package, as shown in **Figure S1**. The variogram line reflects an estimated spherical variogram model, giving the correlation function $\rho(h)$ - a function of the spatial separation vector $h = \|\boldsymbol{s}_i - \boldsymbol{s}_j\|$ and range a:

$$\rho(h) = \begin{cases} 1 - \frac{3h}{2a} + \frac{1}{2}\left(\frac{h}{a}\right)^3 & for\ h \leq a \\ 0 & for\ h > a \end{cases}$$

The estimated nugget, partial sill, and range for each of the outcome were as follows:

Outcome	Variogram parameter	Estimate
Improved Floors	Nugget	0.707
	Partial sill	0.308
	Range (km)	379.44
Improved Walls	Nugget	0.781
	Partial sill	0.249
	Range (km)	262.549
Improved Roofs	Nugget	0.882
	Partial sill	0.283
	Range (km)	498.06

Since the variance of Pearson residuals is known to be 1, the partial sill reflects the proportion of residual variance that is spatially structured. This proportion was as high as 30.8% for improved floors and as low as 24.9% for improved walls. The estimated range was largest for improved roofs (498km) and the smallest for improved walls (263km). Taken together, the variograms reveal non-trivial spatial correlation in the residuals after adjusting for all covariates described in Table 2 of the main text, motivating an explicitly spatial statistical model.

2. *INLA mesh construction*

The INLA "mesh" was constructed on projected coordinates with continental borders treated as a boundary (**Figure S2A**). The maximum edge length defines the precision of the mesh: smaller edges produce more refined meshes. However, we must balance this precision with the associated computational burden. Here, we took the maximum edge to be 155.73km, which is less than the estimated range of the spatial field (from the variograms). The resultant total number of vertices was 18,352. Household locations overlaid over the mesh are shown in **Figure S2B**.

3. INLA statistical model and priors used

All outcome variables in this study were binary; thus, the likelihoods used were Bernoulli with probability $\pi(\boldsymbol{s}_i)$, which necessarily varies with location $\boldsymbol{s}_i$. The INLA latent Gaussian Random Field (GF) models for each outcome were specified as follows:

$$Y(\boldsymbol{s}_i)|u(\boldsymbol{s}_i) \sim Bernoulli(\pi(\boldsymbol{s}_i))$$

$$logit(\pi(\boldsymbol{s}_i)|u(\boldsymbol{s}_i)) = \alpha + x^T(\boldsymbol{s}_i)\boldsymbol{\beta} + u(\boldsymbol{s}_i)$$

$$u(\boldsymbol{s}_1, \dots, \boldsymbol{s}_N) \sim GF[\mathbf{0}, \boldsymbol{\Sigma}_{Matérn}],$$

where $u(\boldsymbol{s}_i)$ is the GF evaluated at location $\boldsymbol{s}_i$, α is the intercept, and $x^T(\boldsymbol{s}_i)$ consists of all fixed effects shown in Table 2 recorded at location $\boldsymbol{s}_i$. The stationary Matérn covariance function $(\boldsymbol{\Sigma}_{Matérn})_{ij} = COV\left(u(\boldsymbol{s}_i), u(\boldsymbol{s}_j)\right)$ is parameterized as follows:

$$COV\left(u(\boldsymbol{s}_i), u(\boldsymbol{s}_j)\right) = \frac{\sigma_u^2}{2^{1-\nu}\Gamma(\nu)}(\kappa h)^\nu K_\nu(\kappa h),$$

where $h = \|\boldsymbol{s}_i - \boldsymbol{s}_j\|$, σ_u^2 is the variance of the GF, κ is a scale parameter, ν is a smoothness parameter, and K_ν is the modified Bessel function of the second kind of order ν. Cameletti and colleagues showed that the practical range $\rho = \sqrt{8\nu}/\kappa$, which is estimated and reported by inlabru, is the distance at which between-location correlation is approximately 0.1 (1).

INLA requires prior distributions be assigned to all hyperparameters of the GF. In our case, the hyperparameters consist of the standard deviation σ_u and (effective) range parameter ρ. Following the recommendations in Simpson and colleagues., as implemented in inlabru (2), we specified penalized complexity (PC) priors, wherein we specify prior probabilities that the standard deviation will exceed a certain value and that the practical range will fail to exceed a certain value. For our data, recalling that we operate on the logit scale for our GF and that our coordinates were rescaled into kilometers, we specified:

$$P(\sigma_u > 3.0) = 0.1 \quad P(\rho < 100) = 0.1.$$

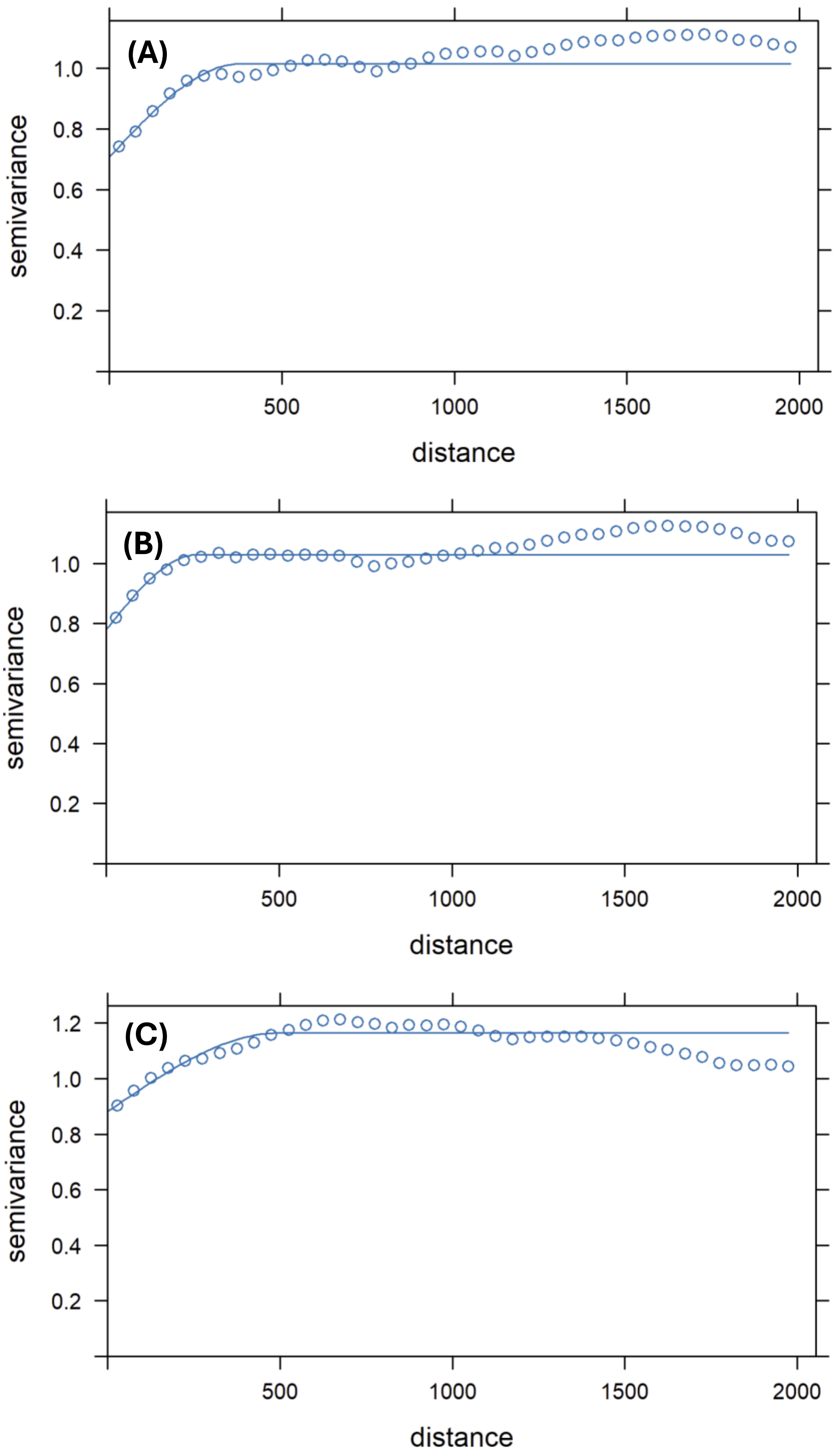

Figure S1: Pearson residual semi-variogram and the estimated spherical model (overlaid line) for improved floors **(A)**, walls **(B)**, and roofs **(C)**. Coordinates were projected via the Mollweide projection and rescaled to kilometers prior to analysis.

(A)

(B)

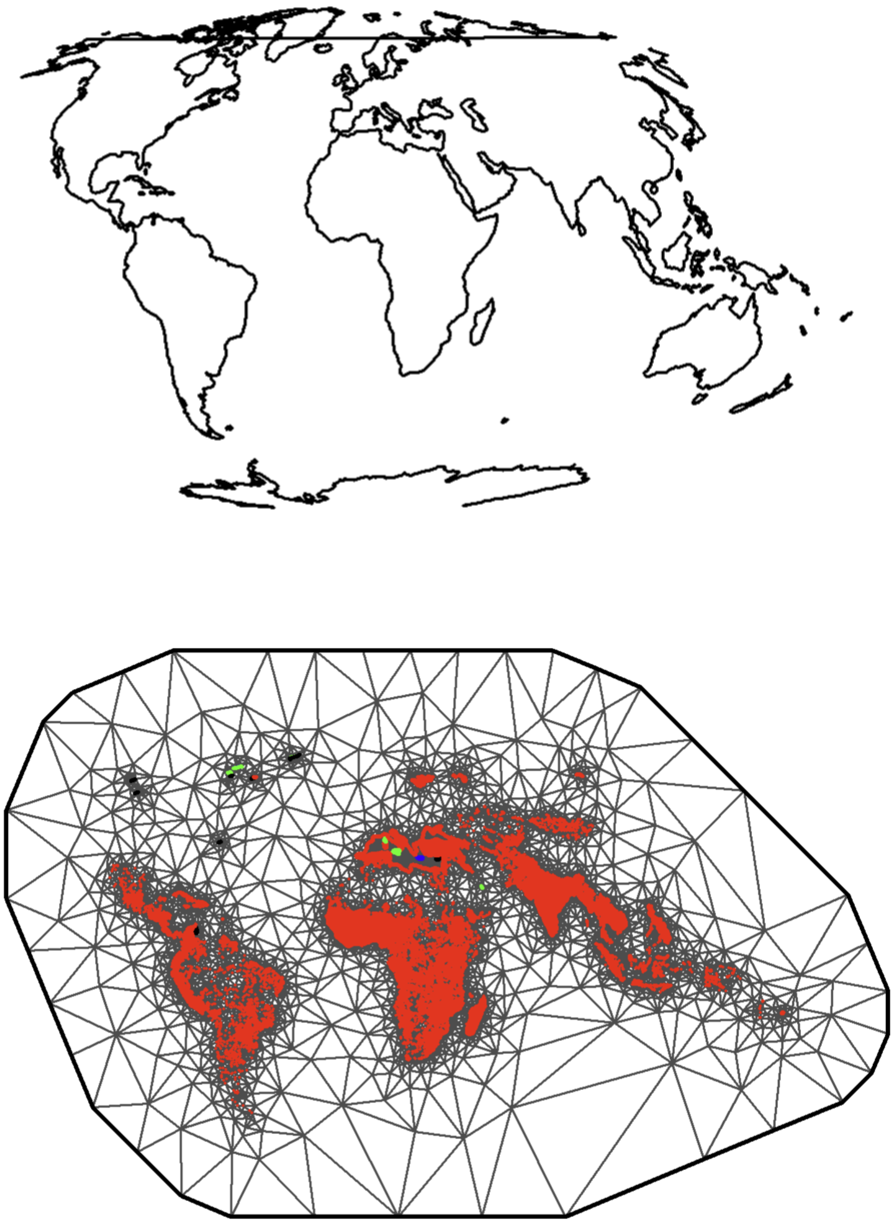

Figure S2: Continental borders used as the boundary for INLA mesh construction. Coordinates and borders were projected via the Mollweide projection prior to analysis **(A)**. INLA mesh with 18352 vertices and locations of households included into the model (red points) **(B)**.

4. *Guideline compliance*

**Table S4: Checklist for Guidelines for Accurate and Transparent Health Estimates Reporting (GATHER) (3)**	
**Objectives and funding**	
1. Define the indicator(s), populations (including age, sex, and geographic entities), and time period(s) for which estimates were made.	Methods - objectives and scope section
2. List the funding sources for the work	Financial support section
**Data inputs**	
3. Describe how the data were identified and how the data were accessed.	Methods section and table 2
4. Specify the inclusion and exclusion criteria. Identify all ad-hoc exclusions.	Methods section
5. Provide information on all included data sources and their main characteristics. For each data source used, report reference information or contact name/institution, population represented, data collection method, year(s) of data collection, sex and age range, diagnostic criteria or measurement method, and sample size, as relevant.	Table 2 and Supplementary File S2
6. Identify and describe any categories of input data that have potentially important biases (e.g., based on characteristics listed in item 5).	Not applicable
7. Describe and give sources for any other data inputs.	Methods – Georeferencing households section
8. Provide all data inputs in a file format from which data can be efficiently extracted (e.g., a spreadsheet rather than a PDF), including all relevant meta-data listed in item 5. For any data inputs that cannot be shared because of ethical or legal reasons, such as third-party ownership, provide a contact name or the name of the institution that retains the right to the data.	Not applicable, see data availability statement.
**Data analysis**	
9. Provide a conceptual overview of the data analysis method. A diagram may be helpful.	Methods –analysis section
10. Provide a detailed description of all steps of the analysis, including mathematical formulae. This description should cover, as relevant, data cleaning, data pre-processing, data adjustments and weighting of data sources, and mathematical or statistical model(s).	Methods –analysis section; Supplementary File S1
11. Describe how candidate models were evaluated and how the final model(s) were selected.	Methods – analysis section
12. Provide the results of an evaluation of model performance, if done, as well as the results of any relevant sensitivity analysis.	Table 3 and figure 4
13. Describe methods of calculating uncertainty of the estimates. State which sources of uncertainty were, and were not, accounted for in the uncertainty analysis.	Methods – analysis section

Table S4: Checklist for Guidelines for Accurate and Transparent Health Estimates Reporting (GATHER) (3)	
14. State how analytical or statistical source code used to generate estimates can be accessed.	See data availability statement. The statistical source code used to generate estimates can be accessed is available from the corresponding author upon reasonable request.
**Results and discussion**	
15. Provide published estimates in a file format from which data can be efficiently extracted.	Supplementary TIFF files of estimates coverage. Supplementary files 3a-c
16. Report a quantitative measure of the uncertainty of the estimates (e.g., uncertainty intervals).	Additional TIFF files giving standard errors of the estimates are provided as Supplementary files 3d-f
17. Interpret results in light of existing evidence. If updating a previous set of estimates, describe the reasons for changes in estimates.	Discussion section
18. Discuss limitations of the estimates. Include a discussion of any modelling assumptions or data limitations that affect interpretation of the estimates.	Discussion section

1. References

1. Cameletti M, Lindgren F, Simpson D, Rue H. Spatio-temporal modeling of particulate matter concentration through the SPDE approach. AStA Adv Stat Anal. 2013 Apr 1;97(2):109–31.

2. Simpson D, Rue H, Riebler A, Martins TG, Sørbye SH. Penalising Model Component Complexity: A Principled, Practical Approach to Constructing Priors. Statistical Science. 2017 Feb;32(1):1–28.

3. Stevens GA, Alkema L, Black RE, Boerma JT, Collins GS, Ezzati M, et al. Guidelines for Accurate and Transparent Health Estimates Reporting: the GATHER statement. The Lancet. 2016 Dec 10;388(10062):e19–23.
