# Supplementary material for: Spatial variation in housing construction material in low- and middle-income countries: A Bayesian spatial prediction model of a key infectious diseases risk factor and social determinant of health
Source: PLOS Glob Public Health. 2024 Dec 18;4(12):e0003338. doi: 10.1371/journal.pgph.0003338 (PMC11654929; doi:10.1371/journal.pgph.0003338)
Supplement: Attachment — Submitted filename: PGPH-D-24-01135 Response to Reviewers.docx [file pgph.0003338.s004.docx]

# PGPH-D-24-01135 – Responses to Reviewer Comments

We thank the reviewers for their helpful reviews. We have addressed each comment below with our response (indented and in italics). As requested, we have provided both a marked-up copy of the amended manuscript as well as an unmarked version with the changes incorporated. Note that in the previous version of the manuscript we identified the wrong corresponding author, and this has now been amended. Responding to this review has significantly improved the manuscript and we ask you to again consider this for publication in *PLOS Global Public Health.*

## Reviewer #1:

This is a well written and well executed study. The figures and background information and scope of work are clear. The figures are consistent with the purpose of the paper.

*We thank the reviewer.*

However, I recommended minor revision because I see a mismatch between the goals of the paper and the conclusions. I recommend that a paragraph be added to the introduction that explicitly states the scientific advancement of the work. Is it the gathering or presenting of the data? Is it the use of this statistical methods? It is not quite clear to the readers. Once this paragraph is explained, then I believe there may be some restructuring needed to emphasize these points. For example, the conclusion section talks about how your statistical approach is fairly computationally efficient. Then I would like a section describing how it is efficient based on its comparison to other methods, including improved background lit review. If the algorithm is critical in this paper, then more information on the formulation of the MCMC algorithm must be supplied. The current explanation is very thin and does not give me enough details to weigh the accuracy of it. Broadly, I like the approach, but it could use more information. Alternatively, if the focus is the implications of the results, then I suggest restructuring the discussion and conclusion sections accordingly.

*This point is well taken. The goals and implications are more related to the results than the statistical methods and we have adjusted the relevant sections accordingly to emphasize this. Incidentally, we did not use an MCMC algorithm because INLA does not require it. INLA instead uses approximations to the posterior distributions that render the analytic approach MCMC-free. This is primarily responsible for the superior computational efficiency.*

## Reviewer #2:

### Overall comments:

This paper presents a new model for coverage of improved floor, roof, and wall components in low- and middle-income countries worldwide. This paper expands on previous research looking at housing materials and separates the information into three components. The paper is very well written, offers great background, has rigorous methods, and has well-presented results.

*We thank the reviewer.*

My recommendations would be to expand the discussion section to a more wholistic analysis of the data as well as to more explicitly describe the importance of the model and how it could be used in the future.

*We have expanded the discussion and conclusion sections to address these aspects.*

### Specific Comments:

#### Introduction

Can the authors provide brief insight into how the improved model is useful? Can it be used to focus intervention efforts, guide government infrastructure plans, etc.?

*We have specified in the final paragraph that the predictions can be used for targeting interventions.*

#### Results

There are some countries shown in Figure 2 which are quite close together but have very different coverages, why do you think that may be occurring? What variables from Figure 4 may be driving the prediction? This could be talked about in the discussion.

*We have added some discussion of what variables might drive between-country variation to the discussion section.*

What are the plot subsets showing in Figure 2?

*They are inset maps to show how the predictions look close up. We have amended the figure legend to explain this.*

#### Discussion

The second paragraph feels like more background/introduction material rather than a discussion.
I would be interested in a more direct discussion of the implication of your results, how they compare to other studies, why they are changing over space, and why the three components may differ from one another.

*We have moved the second paragraph to the background section and added discussion of the implications of our results to address the areas that the reviewer highlighted.*

Are the key drivers of the model from Figure 4 from reliable data sets? Does data reliability or availability vary by country?

*All the covariates are taken from reliable sources that have been peer reviewed and undergone valdiation. Most use earth observation data gathered by satellites as inputs, so they are available for the entire domain of interest and political territories are unlikely to be a source of variation in reliability. The exceptions to this are the two economic covariates, GDP and HDI, the authors of which acknowledge that country-specific differences in reporting of national statistics may be a source of uncertainty.*

#### Conclusion

The authors state the authors would be in a "useable format", but who is it usable to (future researchers, policy makers, or the general public)?

*We have clarified that it’s for researchers, program planners and other stakeholders.*

## Reviewer #3:

### General Comments:

This manuscript reports an innovative and robust spatial analysis of global housing quality, making a clear case for the relevance of the analyzed housing characteristics to global public health. The authors have obtained and harmonized a considerable amount of data across many domains to produce subnational-scale maps of predicted housing quality characteristics that will be highly valuable as inputs to future global health analyses, particularly if the corresponding prediction uncertainties can also be mapped and shared.

*We thank the reviewer.*

The methodology is well-described and sound, but additional discussion of a pair of methodological considerations would be beneficial. First, what are the impacts and implications of the two-stage sampling design in the underlying outcome data for the spatial approach employed? While the original survey data are nationally representative, this analysis does not appear to make use of the survey weights. Furthermore, a single household was randomly selected to represent each cluster. Can the authors comment on whether the survey design/weights and this within-cluster subsampling are relevant to the spatial models used in this analysis and why or why not that is the case?

*It is correct that this analysis did not make use of the survey weights. These weights are used when calculating national or region-level indicator values from individual surveys to account for when complexities of the multi-stage sampling design result in households having unequal probabilities of being included in the sample. They are therefore not relevant to this analysis in which we aimed to model indicators as continuously varying through geographic space across multiple countries and surveys. The purpose of the within-cluster subsampling was to account for the impact of two-stage sampling. Since geolocation occurs at cluster level, multiple households within the same cluster are assigned the same location, but could have different, conflicting outcome values (e.g. improved and unimproved floors within the same cluster). With the subsampling we attempt to ensure that each sampled location has just one outcome value. We have added a sentence to this effect in the “Analysis” section.*

Second, were temporal aspects considered besides the continuous log-months elapsed variable that was used as model predictor? While a full spatiotemporal analysis would likely be infeasible given the scale of the analysis and that the surveys are not really longitudinal, the assumption that a given housing characteristic changed over time in a globally uniform log-linear manner requires some justification. Have I correctly interpreted the model structure, and was there consideration given to allowing the effects of time to vary by region or other characteristic, or to assessing temporal autocorrelation in the empirical semivariogram analysis?

*The reviewer has interpreted the model structure correctly with respect to temporal aspects. It is also true that the data was not structured longitudinally, since each household only contributes one observation at a single time point, thus precluding a full spatiotemporal analysis. Our choice to model time log-linearly* *was informed by the assumption that changes in material used for housing tend to be infrequent and unidirectional – i.e. households seldom change the material used in their construction and when they do it is generally from unfinished to finished and not the other way. Text has been added to further clarify this. We did consider allowing time to vary by region, but this led to implausibly sharp gradients at the borders of adjacent countries that were in neighboring regions.* *We agree with the Reviewer that assessing temporal autocorrelation would be an interesting extension of our work. However, the focus of our paper was on geographic variability using the most up-to-date data (i.e., the most recent snapshot in time) so this would be beyond its scope.*

### Specific Comments:

L97-99: How many households did each cluster generally contain? Consider reporting e.g. the median and IQR households per cluster to provide context for the proportion of observations withheld from the analysis.

*Nationally representative household surveys typically sample 25-30 households per cluster. We have added a citation for this. They do not report the total number of households per cluster from which these are sampled.*

L111: Was there a rationale for using the spherical correlation model? (It’s a perfectly reasonable choice, just curious if there was a specific motivation for choosing it.)

*We used the spherical model in our exploratory analysis to justify the need to account for spatial correlation in the residuals of a model with a rich mean structure. Unlike other common correlation models (e.g., exponential, gaussian, matern), the spherical correlation model returns the range (not the effective range) and so this was the most straightforward way to answer the question “are the residuals correlated in the space after accounting for the mean structure and if so, to what degree?”. This is outlined in supplementary file S1.*

L126: How was the number of mesh vertices selected?

*Ideally, the number of vertices is selected such that: 1) the spatial dimension is sufficiently reduced to enable computation and 2) the number of vertices is such that the maximum edge of the resultant mesh triangles is approximately  1/3 to 1/10 of the range (*[*Bakka et al., 2018*](https://wires.onlinelibrary.wiley.com/doi/10.1002/wics.1443)*). With a spatial prediction task of high dimension, the number of vertices must be balanced with computational costs. We selected the number of vertices such that all global regions of interest were covered and the maximum edge of 155.7km was approximately 1/3 of the model-estimated range. Details on mesh construction are provided in Supplementary Materials along with two figures.*

L127-128: Consider noting in the main text that the INLA spatial models were specified with a Matern covariance function.

*We have noted this in the main text.*

L132-133: Was prediction uncertainty also assessed? As these prediction maps are intended to be used as inputs for future analyses, it would be useful to also have access to a measure of uncertainty, such as companion maps featuring the prediction standard error and/or upper and lower bounds of the e.g. 90% prediction interval at each location.

*We calculated the standard errors for the prediction estimates and provided these as separate supplementary raster files alongside the predictions themselves. Users can calculate prediction intervals for the coverage estimates themselves at any location using these.*

L145-149: If feature importance was assessed simply by scaling the regression coefficients, as the text suggests, why could this not be done using the parameter estimates for the fixed effects from the inlabru models? Was some other technique (e.g., permutation feature importance) from Scikit-learn used?

*We agree with the reviewer’s suggestion. We have amended figure 4 and the assessment of feature importance to use the scaled parameter estimates for fixed effects and have amended the text accordingly.*
